# An Unusual Accessory Muscular Belly of the Flexor Carpi Radialis Muscle

**DOI:** 10.7759/cureus.102386

**Published:** 2026-01-27

**Authors:** George Tsakotos, George Triantafyllou, Alexandros Samolis, Angelos Kandilas, Ioannis Vasilakis, Dionisis Leimonis, Konstantinos Natsis, Maria Piagkou

**Affiliations:** 1 Department of Anatomy, School of Medicine, Faculty of Health Sciences, National and Kapodistrian University of Athens, Athens, GRC; 2 Department of Anatomy and Surgical Anatomy, School of Medicine, Faculty of Health Sciences, Aristotle University of Thessaloniki, Thessaloniki, GRC

**Keywords:** accessory bundle, dissection, flexor carpi radialis muscle, forearm, radial nerve, variation

## Abstract

Variations of the upper limb musculature are of clinical interest, potentially impacting diagnostic interpretation and surgical outcomes. This report describes a rare anatomical variation identified in a 72-year-old male cadaver, i.e., an accessory flexor carpi radialis (FCR) originating from the medial border of the brachioradialis (BR). While current classifications categorize FCR variants based on origins from the bicipital aponeurosis, biceps brachii, or pronator teres, this case represents a unique morphology. Embryologically, such bundles likely arise from the incomplete separation of the common flexor-pronator mass during morphogenesis. Clinically, these accessory bellies may be misidentified as soft tissue tumors on imaging or complicate surgical procedures like tendon transfers and volar approaches to the radius. Furthermore, their proximity to the superficial branch of the radial nerve creates potential sites for nerve compression and entrapment syndromes.

## Introduction

Variations of the upper limb musculature are of considerable anatomical and clinical interest due to their potential implications for diagnostic interpretation, surgical planning, and neurovascular integrity.

Accessory muscular belies of the forearm have been adequately described in the current literature. Most common forms correspond to the Gantzer muscle [1], the Linburg-Comstock connection [2], the accessory brachialis bellies [3], the accessory brachioradialis muscle [4], and other innominate variants [5,6].

The flexor carpi radialis (FCR) muscle typically originates from the medial epicondyle of the humerus and inserts into the bases of the second and third metacarpal bones and functions as a principal wrist flexor and radial deviator. Variations affecting its distal course or insertion are uncommon but clinically relevant, as they may modify forearm biomechanics or establish atypical relationships with neighboring musculotendinous or neurovascular structures [7].

During their cadaveric study, Zhou et al. [7] proposed a classification system of FCR variants. They proposed four types of accessory FCR (aFCR) forms. In this context, we are describing an unusual aFCR case identified during cadaveric dissection.

## Case presentation

During the routine dissection of the right upper limb of a 72-year-old Greek donated male cadaver, an unusual muscular variation was identified. No evidence of previous surgical intervention or traumatic injury was observed. Following removal of the skin and superficial fascia, the anterolateral compartment of the forearm was exposed.

**Figure 1:**
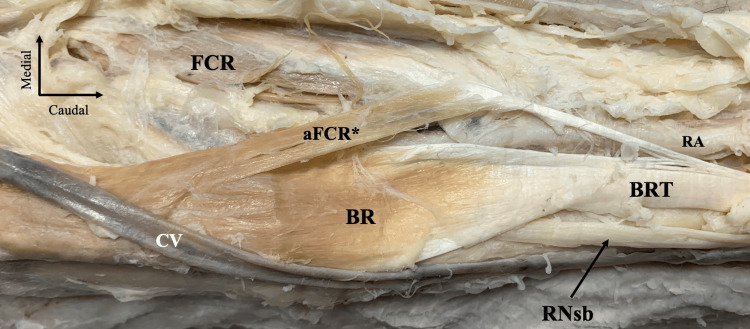
Dissection of the right forearm region. The brachioradialis (BR) muscle is located in the anterior compartment of the forearm, along with the cephalic vein (CV). An accessory muscular belly is originating from the BR distal part, and it is inserting into the flexor carpi radialis (FCR) muscle tendon. Therefore, it was considered an accessory belly of the FCR (aFCR*). The superficial branch of the radial nerve (RNsb) is typically located laterally to the BR tendon (BRT), and the radial artery (RA) medially.

The brachioradialis (BR) was identified in its typical anatomical position, forming the principal muscular mass along the lateral aspect of the forearm. Superficially, the cephalic vein coursed longitudinally along the lateral margin of the dissection field. A distinct accessory muscular structure originated from the medial border of the BR. This accessory belly coursed obliquely and distally, lying superficial and medial to the main BR belly. Proximally, it exhibited a muscular configuration, while distally it gradually transitioned into a tendinous component. Distally, the muscular belly attached to the FCR; therefore, it was considered an accessory muscular belly of the muscle (Figure 1). Distally, the BR tendon was attached to the lateral surface of the distal radius, immediately proximal to the radial styloid process. The radial artery was identified anterior to the BR tendon, while the radial nerve superficial branch coursed nearby, consistent with the typical distal anatomical relationships of these structures.

## Discussion

Integrating the current findings with established anatomical literature, the observed variation represents a rare accessory muscular bundle of the FCR. Such accessory bundles are uncommon, with five cases previously reported [7,8]. Recent morphological classification categorizes FCR variations into four distinct types (A-D) based on the accessory bundle's origin and its relationship with surrounding structures like the bicipital aponeurosis, biceps brachii, and pronator teres [7]. Type A corresponds to the accessory head that arises from the distal part of the bicipital aponeurosis. Types B and D correspond to the bundle that connects with the pronator teres or its own accessory structures. Type C corresponds to the bundle that originates from the biceps brachii covered by the bicipital aponeurosis [7]. Thus, the current case corresponds to an unusual accessory FCR form (originating from the BR) that cannot fit into any type reported by Zhou et al. [7]. The presence of these accessory heads often co-occurs with other muscular anomalies in the forearm, suggesting a pattern of concomitant variations [7]; however, we did not observe a coexisting variant in the current case.

Similar supernumerary heads of the FCR have been observed in non-human primates and other mammals, indicating a potential comparative anatomical basis for these human variants [7]. Moreover, the occurrence of an accessory FCR bundle can be explained through early limb morphogenesis. During embryonic development, the forearm muscles originate from a common flexor muscular mass and typically differentiate and separate from the proximal to the distal pole. Variations like the one described likely result from the insufficient or incomplete separation of this pre-muscle mass during early stages [7,9].

Even though exceedingly uncommon, the clinical significance of an accessory FCR is multifaceted. From a diagnostic perspective, these accessory muscular bundles may be encountered radiologically or intraoperatively [7]. On imaging, an aFCR can be misidentified as a soft tissue mass or tumor, making magnetic resonance imaging and ultrasonography essential tools for differentiating anatomical variants from pathologies such as ganglia or fibromas [7,8].

Surgically, the FCR is a common donor for tendon transfer procedures, particularly in treating radial nerve palsy; however, the presence of a variation must be evaluated in advance to prevent suboptimal postoperative effects [10]. Surgeons utilizing a volar approach must maintain awareness of such anomalies to avoid selecting a false surgical plane or causing iatrogenic injury [11]. Furthermore, the close anatomical relationship between an accessory FCR and the radial nerve branches creates potential sites for nerve compression. Variations arising near the BR can narrow the neurovascular intervals, potentially leading to superficial radial nerve compression at the elbow, characterized by sensory deficits in the dorsal thumb and hand [10]. Such anomalous muscles are documented causes of cheiralgia paresthetica, or Wartenberg’s syndrome, where the nerve is pinched between muscular or tendinous slips, resulting in chronic pain and dysesthesia [10].

## Conclusions

The current case reports a unique accessory FCR originating from the BR, a morphology that expands upon the previously proposed four-type classification system. From a developmental perspective, this variation likely represents a residual muscular slip resulting from the incomplete separation of the forearm’s common flexor-pronator mass during early morphogenesis. The clinical importance of the accessory FCR bundles lies in diagnostic awareness, surgical precision, and neurovascular compression.

## References

[1] Asghar A, Jha RK, Patra A, Chaudhary B, Singh B (2022). The prevalence and distribution of the variants of Gantzer's muscle: a meta-analysis of cadaveric studies. Anat Cell Biol.

[2] Yurasakpong L, Meemon K, Suwannakhan A (2018). Linburg-Comstock variation: histoanatomy and classification of the connection between flexor pollicis longus and flexor digitorum profundus to the index finger. Surg Radiol Anat.

[3] Piagkou M, Triantafyllou G, Koutsougeras A (2024). A bilateral four-headed brachialis muscle with a variant innervation: a cadaveric report with possible clinical implications. Surg Radiol Anat.

[4] Triantafyllou G, Koptas K, Zielinska N, Piagkou M, Olewnik Ł (2024). The accessory brachioradialis muscle: prevalence of a rare variant with possible clinical implications. Surg Radiol Anat.

[5] Triantafyllou G, Koptas K, Zielinska N, Piagkou M, Olewnik Ł (2025). The brachioradialis longus: an unreported accessory form of the brachioradialis muscle. Anat Sci Int.

[6] Gabríková K, Kachlík D, Belbl M, Kunc V (2023). An accessory muscle belly or an accessory muscle head? An unusual arrangement of muscles in the anterior compartment of the forearm. Surg Radiol Anat.

[7] Zhou M, Ishizawa A, Akashi H, Suzuki R, Bando Y (2023). Morphologic observation for anomalous patterns of the flexor carpi radialis muscle and atypical insertions: a proposal for a new classification. Anat Sci Int.

[8] Zhou M, Ishizawa A, Akashi H, Suzuki R, Kanatsu Y, Abe H, Bando Y (2020). An anomalous case of the flexor carpi radialis with an excessive muscular bundle. Anat Sci Int.

[9] Bardeen CR (1905). Studies of the development of the human skeleton. Am J Anat.

[10] Węgiel A, Karauda P, Zielinska N, Tubbs RS, Olewnik Ł (2023). Radial nerve compression: anatomical perspective and clinical consequences. Neurosurg Rev.

[11] Kravarski M, Goerres GW, Antoniadis A, Guenkel S (2020). Supernumerary brachioradialis - anatomical variation with magnetic resonance imaging findings: a case report. World J Orthop.

